# Novel Clinical, Immunological, and Metabolic Features Associated with Persistent Post-Acute COVID-19 Syndrome

**DOI:** 10.3390/ijms25179661

**Published:** 2024-09-06

**Authors:** Karina Santana-de Anda, Jiram Torres-Ruiz, Nancy R. Mejía-Domínguez, Beatriz Alcalá-Carmona, José L. Maravillas-Montero, José Carlos Páez-Franco, Ana Sofía Vargas-Castro, Jaquelin Lira-Luna, Emmanuel A. Camacho-Morán, Guillermo Juarez-Vega, David Meza-Sánchez, Carlos Núñez-Álvarez, Marina Rull-Gabayet, Diana Gómez-Martín

**Affiliations:** 1 Immunology and Rheumatology Department, Instituto Nacional de Ciencias Médicas y Nutrición Salvador Zubirán, Mexico City 14080, Mexico; karina.santana@incmnsz.mx (K.S.-d.A.); jiram.torresr@incmnsz.mx (J.T.-R.); beatriz.alcalac@gmail.com (B.A.-C.); nuac80df@gmail.com (C.N.-Á.); rull.marina@gmail.com (M.R.-G.); 2 Red de Apoyo a la Investigación, Instituto Nacional de Ciencias Médicas y Nutrición Salvador Zubirán, Mexico City 14080, Mexico; nmejia@cic.unam.mx (N.R.M.-D.); maravillas@cic.unam.mx (J.L.M.-M.); paez@cic.unam.mx (J.C.P.-F.); guillermovega@cic.unam.mx (G.J.-V.); dmeza@cic.unam.mx (D.M.-S.); 3 Hospital Angeles Pedregal, Mexico City 10700, Mexico; asofivargas@gmail.com; 4 Departamento de Cirugía General, Hospital Regional 1ro de Octubre, ISSSTE, Mexico City 07760, Mexico; jaquelin.lira.luna@gmail.com; 5 Departamento de Medicina Crítica, Instituto Nacional de Perinatología, Isidro Espinosa de los Reyes, Mexico City 11000, Mexico; abimelecamacho@gmail.com

**Keywords:** persistent PACS, B cells, monocytes, neutrophils, chemokines, metabolomics

## Abstract

The coronavirus disease 2019 (COVID-19) survivors are frequently observed to present persistent symptoms constituting what has been called “post-acute COVID-19 syndrome” (PACS) or “long COVID-19”. Some clinical risk factors have been identified to be associated with PACS development; however, specific mechanisms responsible for PACS pathology remain unknown. This study investigates clinical, immunological, and metabolomic risk factors associated with post-acute COVID-19 syndrome (PACS) in 51 patients, assessed 7–19 months after acute infection. Among the participants, 62.7% were male and 37.2% were female, with an average age of 47.8 years. At the follow-up, 37.2% met the criteria for PACS, revealing significant differences in immunological and metabolomic profiles at the time of acute infection. Patients with PACS were characterized by elevated levels of mature low-density granulocytes (LDGs), interleukin-8 (IL-8), pyruvate, pseudouridine, and cystine. Baseline multivariate analysis showed increased pyruvate and decreased alpha tocopherol levels. At follow-up, there was a decrease in absolute B lymphocytes and an increase in non-classical monocytes and 3-hydroxyisovaleric acid levels. These findings suggest that specific immunological and metabolomic markers during acute infection can help identify patients at higher risk of developing persistent PACS.

## 1. Introduction

The coronavirus disease 2019 (COVID-19) pandemic has affected millions of patients worldwide; multisystemic involvement due to severe acute respiratory syndrome coronavirus 2 (SARS-CoV-2) has been well described; however, survivors are frequently observed to present persistent neurological, respiratory or cardiovascular symptoms, constituting what has been called “post-acute COVID-19 syndrome” (PACS) or “long COVID-19” that potentially lasts for weeks or months [1], the evidence mostly limited to the first year post infection. Few studies with longer follow-ups assessed limited symptoms or focused exclusively on neurologic sequelae [2,3,4].

The World Health Organization (WHO) defines it as a condition characterized by symptoms impacting everyday life, such as fatigue, shortness of breath, and cognitive dysfunction, which occur after a history of probable or confirmed SARS-CoV-2 infection [5].

Some clinical risk factors have been identified to be associated with PACS development, among these are asthma, type 2 diabetes mellitus, obesity, preexisting clinical depression, hypothyroidism, severe COVID-19 that required hospitalization, and other symptoms during the acute infection [6].

Several studies have been initiated to identify factors associated with the development of PACS; however, specific mechanisms responsible for PACS pathology remain unknown. Cellular damage, a robust innate immune response with inflammatory cytokine production, and a pro-coagulant state induced by SARS-CoV-2 infection may contribute to these sequelae [7,8,9]. It has been reported that PACS can affect all kinds of COVID-19 patients, independently of the acute infection severity, but is more frequent in patients who suffered severe or critical disease requiring hospitalization in comparison with those with mild disease [10,11].

Based on the recent literature, PACS can be divided into two categories: subacute or ongoing symptomatic COVID-19, which includes symptoms and abnormalities present from 4–12 weeks beyond acute COVID-19, and chronic or post-acute COVID-19 syndrome, which includes symptoms and abnormalities persisting or present beyond 12 weeks after the onset of acute COVID-19 and not attributable to alternative diagnoses [12,13].

The pathogenesis of PACS is unknown; however, immunologic abnormalities and inflammatory damage are potential factors involved [14]. Within the immunological abnormalities that have been described in the T-cell compartment include low percentage of näive, central memory, effector memory, and terminally differentiated CD4+ T cells [15]. Previous evidence supports the role of an abnormal immune response in the development of PACS. Klein et al. reported diverse changes in immune factors. They identified, among patients with PACS, some immune cell population changes when compared with the non-PACS population such as increased non-conventional monocytes, double-negative B cells, and IL-4/IL-6 secreting CD4+ T cells.

Monocytes are divided based on the presence of cell surface lipopolysaccharide (LPS) co-receptor CD14 and Fc gamma receptor III (CD16). Classical monocytes account for approximately 85% of circulating monocytes, intermediate cells account for 5%, and non-classic monocytes constitute 10%. Each subset differs in the ability to secrete cytokines and response to pathogen-associated molecular patterns (PAMPs). Classical monocytes are phagocytic and secrete TNFα, but less of other proinflammatory molecules [16]. Recently, a study reported that intermediate and non-classical monocytes were significantly elevated in patients with PACS; in addition, SARS-CoV-2 S1 protein was detected in non-classical monocytes, suggesting that this cell subtype may contribute to inflammation in PACS [17].

A growing body of literature implicates neutrophils as drivers of the hyperinflammatory state during the acute phase of COVID-19; moreover, neutrophil extracellular traps (NETs) are known to contribute to promoting a hyperinflammatory state during COVID-19. This combined evidence demonstrates that excessive neutrophils and NET production is associated with increased disease severity, pathophysiology, and poor clinical outcomes in COVID-19 [18].

Low-density granulocytes (LDGs) are a subpopulation of neutrophils with an enhanced ability to form NETs known to contribute to immune-mediated pathology through proinflammatory cytokine production and in the context of acute COVID-19 infection. Frequencies of LDGs in patients with mild to severe disease are increased compared with healthy controls [19]. Although elevated levels of LDGs are thought to play an important role in disease severity in COVID-19 patients, their circulating levels and functional contributions to long-term effects of COVID-19 and PACS development are unknown.

Globally, the number of patients recovering from COVID-19 infection continues to grow at an unprecedented rate. To date, it is unknown whether and over what time the risk of PACS may be attenuated or became not clinically significant; there are limited data regarding PACS beyond 1 year after the acute infection.

This study is focused on identifying clinical, immunological, and metabolomic factors associated with the persistence of PACS 7–19 months after the acute COVID-19 infection.

## 2. Results

Among the 51 patients included, 32 (62.7%) were males and 19 (37.2%) females with an average age of 47.8 years old. The most common comorbidities observed were obesity (29.4%), systemic arterial hypertension (25.4%), and type 2 diabetes mellitus (15.6%). Regarding acute COVID-19 severity at admission, 21 (41.17%) patients were classified as having mild to moderate COVID-19, 26 (50.9%) patients had severe and 4 (7.8%) had critical conditions. At the follow-up visit, 19 subjects (37.2%) fulfilled PACS classification criteria—6 (28.5%), 11 (42.3%), and 2 (50%) patients from each group, respectively.

From the 19 patients who developed persistent PACS as defined in the Methods section, 12 (63.2%) were males and 7 (36.8%) were females.

Among patients with persistent PACS, the most frequent symptoms we found were cephalea (57.8%), arthralgias (47.3%), as well as respiratory symptoms, such as cough (36.8%) and dyspnea (36.8%) (Supplementary Table S1).

In Table 1, Table 2 and Table 3, baseline demographic, clinical, immunological, and metabolomic features are shown. There were no significant differences on demographic, clinical, immunological, and metabolomic features, other than of those shown at the tables. We found that patients who developed persistent PACS were characterized, at baseline, by an elevated percentage of mature LDGs and IL-8 serum levels. In terms of the metabolomic profile, those patients showed increased pyruvate, pseudouridine, and cystine serum levels.

Interestingly, when we analyzed the severity of the acute infection, we found, notably, that IL-8 and low-density granulocyte levels were significantly lower in the mild group than in the moderate group (*p* = 0.003359 and *p* = 0.006957, respectively). However, the severity of acute infection did not have a significant effect on persistent PACS (*p* = 0.2796). Values in bold reflect statistical significance.

Based on the previous data, we performed univariate and multivariate analyses at baseline and at the follow-up visit to address the immunological and metabolomic features associated with the presence of PACS at 7–19 months after the acute SARS-CoV-2 infection.

In the univariate analysis, the variables associated with the presence of persistent PACS were BMI (OR 1.5, 95% CI 1.004–1.352), *p* = 0.04, ALT (OR 0.99, 95% CI 0.983–0.999), *p* = 0.01, pyruvate (OR 1.83, 95% CI 1.096–3.642), *p* = 0.01, cystine (OR 1.15, 95 CI 1.021–1.365), *p* = 0.01, alpha tocopherol (OR 0.59, 95% CI 0.309–0.987), *p* = 0.04 (Table 4).

**Figure 1:**
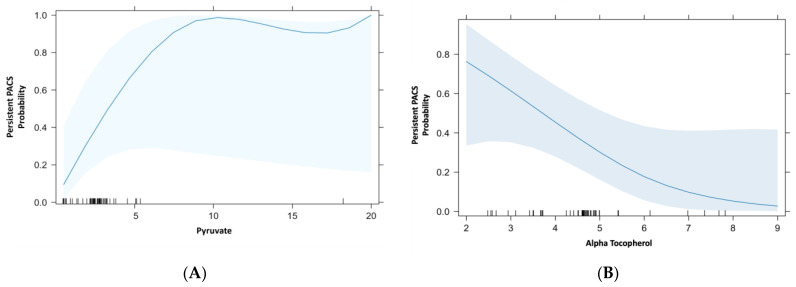
Baseline variables associated with persistent PACS. Multivariate analysis. (**A**) Piruvate: OR 1.99 (95% CI 1.02–4.65) *p* = 0.04. (**B**) Alpha tocopherol: OR 0.5 (95% CI: 0.23–0.56) *p* = 0.03.

In the multivariate analysis (Figure 1), the variables associated with the presence of persistent PACS were increased pyruvate serum levels and decreased alpha tocopherol serum levels.

**Figure 2:**
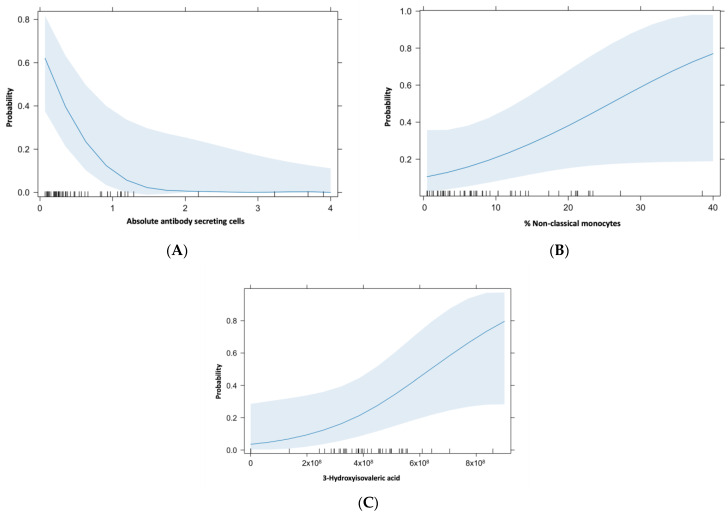
Variables present among patients with persistent PACS. Multivariate analysis. (**A**) Absolute antibody secreting cells: OR = 0.05 (95% CI: 0.002–1.21) *p* = 0.001. (**B**) Non-classical monocytes %: OR = 1.08 (95% CI: 1.00–1.2) *p* = 0.04. (**C**) 3-hydroxyisovaleric acid: OR = 1 (95% IC: 1.00–1.00), *p* = 0.01.

In the multivariate analysis at the follow-up visit (Figure 2), interestingly, we found an association of an increased percentage of non-classical monocytes, decreased absolute numbers of antibody secreting cells, and increased 3-hydroxyisovaleric acid serum levels (OR 1, 95% CI 1.00–1.00), *p* = 0.01 among patients with persistent PACS.

Finally, when we evaluated the effect of temporal change of clinical, metabolomic, and immunological variables, we found a tendency of an increased absolute numbers of non-classic monocytes among patients with persistent PACS.

## 3. Discussion

The main findings of our study are that higher serum levels of pyruvate and lower serum levels of alpha tocopherol at the time of SARS-CoV-2 acute infection are risk factors for PACS development at 7 months and beyond. Among patients with PACS, at the follow-up time, we found increased percentage of non-classic monocytes, decreased absolute antibody secreting cells, and increased 3-hydroxyisovaleric acid at the time of the follow-up evaluation. Additionally, when we analyzed the effect of temporal change among the different variables, we confirmed increased numbers of non-classic monocytes among patients with persistent PACS.

Due to the high prevalence reported, PACS is becoming an important public health issue. In that sense, it is of great importance to understand and identify at an early stage of the acute infection those patients at higher risk of PACS development.

A comprehensive understanding of the host from the clinical, immunologic, and metabolomic perspective in COVID-19 individuals who develop PACS is important for developing early therapeutic treatments or interventions.

Some clinical risk factors have been identified, including female sex, older age, some comorbidities as diabetes mellitus, obesity, the number of symptoms during the acute infection, and the diagnosis of severe COVID-19 [10,20,21]; some of them are corroborated in our study, as diabetes mellitus and the diagnosis of severe COVID-19.

In our cohort, we found an elevated percentage of mature LDGs at baseline among the patients who developed PACS in comparison of those who did not develop the outcome; it has been described that LDGs play an important role in disease severity in COVID-19 patients [18]. Recently, it has also been described that circulating LDGs remain elevated during PACS onset, specifically mature LDGs [22]; they are also associated with more severe forms of the disease [23]. In our cohort, we found an elevated percentage of mature LDGs at the time of the acute infection but not at the follow-up visit. However, as mature LDGs are elevated at the time of the acute infection, this may be in relation with the severity of the disease and must be analyzed in further studies.

At the follow-up visit, 7 months and beyond after the acute infection, we found an increased percentage of non-classical monocytes, which agrees with what previous literature reports have mentioned, demonstrating that intermediate and non-classical monocytes are significantly elevated in patients with PACS [17,24]. This finding highlights that COVID-19 convalescents have monocyte dysregulation beyond the acute infection; monocytes have emerged as key cellular modulators of COVID-19 pathophysiology. Also, monocyte dysregulation has been associated with favoring SARS-CoV-2 virus persistence in some tissues outside the respiratory tract. For example, the brain demonstrated virus replication in multiple non-respiratory sites during the first two weeks following symptom onset and detected subgenomic RNA in at least one tissue in 14 of 27 cases beyond day 14, indicating that viral replication may occur in non-respiratory tissue for several months [25]. These data may explain some of the pathology mechanisms of persistent PACS development. Few studies have assessed the immune profiles in patients with PACS; however, it is still unknown whether immune cell dysregulation contributes to the physiopathology of PACS.

There is scarce information regarding the metabolomic profile at the acute SARS-CoV-2 infection as well as during PACS. In 2022, Guntur V et al. [26] reported, for the first time, metabolic derangements in patients with PACS, providing new insights into the potential role of metabolism in the pathogenesis of this condition. The group reported that patients who developed PACS exhibit lactate accumulation and respiratory gas exchange indicative of impaired fatty acid oxidation during exercise challenge, suggesting mitochondrial dysfunction [27]. These results support the hypothesis that a disfunction in substrate utilization in mitochondria underlies the metabolic manifestations of PACS and that metabolomic alterations found in acute COVID-19 infection may persist in those who develop PACS; this group compared healthy individuals, patients who fully recovered from acute SARS-CoV-2 infection without PACS, and patients with PACS corroborating significantly higher levels of plasma pyruvate in patients with PACS. In our cohort, we identified increased levels of pyruvate at the time of the acute infection as a risk factor to develop PACS. This finding may support the hypothesis of mitochondrial dysfunction as part of the PACS pathophysiology as well as the elevated levels of 3-hydroxyisovaleric acid among patients with PACS we found, consistent with the lower fatty acid oxidation capacity of mitochondria. Recently, Lopez-Hernandez et al. demonstrated that lactate and pyruvate are positively correlated with fatigue, myalgia, and arthralgias, highly suggesting that these two metabolites and the mitochondrial disfunction may be importantly implicated in the pathology of persistent PACS [28].

In conclusion, this is the first study, to the best of our knowledge, in which clinical, immunological, and metabolomic features in patients with acute COVID-19 were assessed all together and correlated with the presence of persistent PACS.

PACS pathophysiology is complex and not well understood. Our study, as well as previous reports, show immunological and metabolomic dysfunction. Early detection of patients at risk of PACS development must be identified and therapeutic interventions performed. The wide variety of immunological and metabolomic dysfunctions early in the acute infection may persist for several months and explain the highly heterogeneous clinical presentation of persistent PACS. This is the reason why it is important to start classifying persistent PACS patients according to clinical, immunological, and metabolomic profiles.

Our study has several limitations including a small sample size and that the length of time between acute active SARS-CoV-2 infection and the follow-up visit was variable, ranging from 7 to 19 months. This variability in time of sample collection may have influenced some immunological and metabolomic features. Other potential confounders are that we did not have information regarding re-infections between the initial acute infection and the follow-up visit, as well as the vaccination status and number and type of vaccines received by each subject. Further studies are needed with serial assessment of clinical, immunological, and metabolic features over the time between the acute infection and the development and follow-up of PACS.

## 4. Materials and Methods

A cohort study of 51 patients with acute SARS-CoV-2 infection confirmed by the nasopharyngeal swab polymerase chain reaction (PCR) test was conducted between 1 August 2021 and 28 February 2022 at Instituto Nacional de Ciencias Médicas y Nutrición Salvador Zubirán (INCMNSZ) in Mexico City, a referral center for this entity. This study was approved by the Institutional Ethics and Research Committees (REF 3734) according to the Helsinki declaration, and all patients signed an informed consent prior to their inclusion.

The patient baseline evaluation was performed at the first-time visit to the INCMNSZ emergency department; all the patients underwent complete clinical evaluation, laboratory, and radiologic assessment. Prior to the administration of any treatment, a blood sample was drawn at baseline to obtain laboratory tests; additionally, serum and plasma samples were stored at −80 °C until further analysis. All patients were stratified according to disease severity as shown below [7]:

(A) Mild/moderate disease: fever, upper respiratory infection symptoms, with or without pneumonia.
(B) Severe: Any of the following: respiratory failure, respiratory rate >30 breaths per minute, oxygen saturation at rest < 93%, PaO_2_/FIO_2_ < 300 mmHg.
(C) Critical: any of the following: requirement of invasive mechanical ventilation, shock, multiple organ failure.

PACS was diagnosed in patients with fatigue and at least two of the multiorgan symptoms previously described [13]. Symptoms were evaluated using a standardized questionnaire used in one of our previous cohorts [15].

The following experimental procedures were performed at baseline and within a follow-up visit (7–19 months after the acute infection).

### 4.1. Peripheral Blood Mononuclear Cells (PBMCs) Immunophenotyping by Flow Cytometry

PBMCs were isolated by density gradients with Ficoll-Paque (GE Healthcare Life Sciences, Chicago, IL, USA). The cells, after two washes with phosphate buffered saline (PBS), were stained with the viability marker Zombie Aqua (Biolegend, San Diego, CA, USA). After washing the cells twice with 5% fetal bovine serum (FBS), the cells were incubated for 30 min at room temperature with FcX blocker (Biolegend, San Diego, CA, USA) and the following fluorochrome-coupled antibodies: CD19-BUV496 (cat. 612938, BD Biosciences, Franklin Lakes, NJ, USA), CD3-APC/Fire-750 (cat. 344840), CD4-Alexa fluor 488 (cat. 317420), CD8-PE/Dazzle-594 (cat. 344744), CD10-PE (cat. 312204), CD11c-PE/Dazzle-594 (cat. 337228), CD14-PerCP (cat. 325632), CD15-FITC (cat. 301904), CD16-Alexa fluor 700 (cat. 302026), CD21-Alexa fluor 700 (cat. 354918), CD24-BV421 (cat. 311122), CD25-BV421 (cat. 302630), CD27-APC-Cy7 (cat. 356424), CD38-BV650 (cat. 356620), CD45RA-PE (cat. 304108), CD45RO-FITC (cat. 304242), CD56-PE (cat. 318306), CD62L-PE/Cy5 (cat. 304808), CD127-BV650 (cat. 351326), CD335-BV650 (cat. 331927), CD355-APC (339108), CCR7-PE/Dazzle-594 (cat. 353236), IgD-PerCP/Cy5.5 (cat. 405710). All of the mentioned antibodies were obtained from Biolegend, San Diego, CA, USA. For the T-helper and cytotoxic subset evaluation, PBMCs were stimulated with phorbol 12-myristate 13-acetate (PMA), ionomycin, and monensin for 5 h at 37 °C. The cells were fixed and permeabilized with the cytofix/cytoperm fixation–permeabilization kit (BD Biosciences, Franklin Lakes, NJ, USA). Intracytoplasmic cytokines were analyzed with fluorochrome-coupled antibodies: IFN-γ-APC (cat. 506510), IL-4-PE (cat. 500810), IL-17-BV421 (cat. 512322) from Biolegend, San Diego, CA, USA. We acquired one million events in a 4-laser LSR Fortessa flow cytometer (BD Biosciences, Franklin Lakes, NJ, USA).

The absolute number of LDG, monocyte, and lymphocyte subsets were calculated with the amount of total leukocytes, monocytes, and lymphocytes in a complete blood count performed on the same day of PBMC isolation. The gating strategy is depicted in Supplementary Figure S1.

### 4.2. Metabolomic Assessment

Serum untargeted metabolomics analysis was performed using gas chromatography coupled to mass spectrometry (GC/MS). A total number of 46 metabolites were detected with relative standard deviation (RSD) < 30% in the quality control (QC) sample, as described previously [29].

### 4.3. Cytokine/Chemokine and Coagulation Profiles Assessment

A total if 32 cytokines and chemokines were measured in plasma using the MILLIPLEX Multi-analyte Profiling (MAP) human cytokine/chemokine magnetic bead panel 29-plex kit (EMD Millipore, Darmstadt, Germany), the TGF-β base magnetic Luminex performance Assay 3-plex kit (R&D systems, Minneapolis, MN, USA), and the ProcartaPlex Multiplex Immunoassay Human Coagulation Panel 4-plex kit (Thermo Fisher Scientific, Waltham, MA, USA) on a 2-laser Bio-Plex 200 suspension array system coupled to a Bio-Plex Pro Wash Station (Bio-Rad, Hercules, CA, USA). Bead-fluorescence intensity readings for all the samples and standards were converted into the corresponding analyte concentrations using the Bio-Plex Manager software v6.2 (Bio-Rad, Hercules, CA, USA).

Within the analytes measured, we included IL-1α, IL-1β, IL-1RA, IL-2, IL-3, IL-4, IL-5, IL-6, IL-7, IL-8, IL-10, IL-12p40, IL-12p70, IL-13, IL-15, IL-17A, IP-10, MCP1, MIP-1α, MIP-1β, TNF-α, TNF-β, VEGF, Eotaxin/CCL11, TGF-β1, TGF-β2 and TGF-β3.

IL-8 serum levels were evaluated by ELISA (MBL, Tokyo, Japan).

All the patients included were re-evaluated 7–19 months after the acute COVID-19 infection. We performed a standardized questionnaire to assess the presence or absence of PACS. This questionnaire was validated previously by our group [15]. The systems addressed were respiratory, cardiovascular, neurological, gastrointestinal, musculoskeletal, psychiatric, ear, nose and throat, and dermatological. At this time, a second blood sample was drawn to collect plasma and serum from each patient which were stored at −80 °C until further analysis. All the immunological and metabolomic features were evaluated at the time of the acute COVID-19 infection and at the follow-up visit.

All patient samples were processed by investigators blinded to the presence or absence of PACS at the time of the follow-up visit.

The primary objective of the present study was the presence or absence of PACS at the follow-up visit defined as the presence of fatigue and at least two of the multiorgan symptoms previously described [13] and to identify clinical, immunological, and metabolomic factors associated.

### 4.4. Statistical Analysis

To address differences between quantitative variables that were expressed as medians and interquartile ranges (IQRs), we employed the U-Mann–Whitney test for binomial variables and performed a G-test. To examine the effect of clinical and immunological variables on the occurrence of post-acute COVID-19 syndrome, univariate and multivariate logistic regression analyses were performed. In multivariate logistic regression analysis, we included variables with *p*-value < 0.1 and performed stepwise selection variables to obtain the best supported multivariate model using minimum Akaike information criteria (AICs). Also, we included the FANSYPOSTCOV index [15] in logistic regression analysis as an explanatory variable to test their predictive ability for post-acute COVID-19 syndrome. Finally, we evaluated the effect of temporal change in clinical and immunological variables on post-acute COVID-19 syndrome by generalized linear mixed model. All statistical analyses were performed using R project software (version 4.3.1).

## 5. Conclusions

In conclusion, this is the first study, to the best of our knowledge, in which clinical, immunological, and metabolomic features in patients with acute COVID-19 were assessed all together and correlated with the presence of persistent PACS.

PACS pathophysiology is complex and not well understood. Our study, as well as previous reports, show immunological and metabolomic dysfunction. Early detection of patients at risk of PACS development must be identified and therapeutic interventions performed. The wide variety of immunological and metabolomic dysfunctions early in the acute infection may persist for several months and explain the highly heterogeneous clinical presentation of persistent PACS, which is why it is important to start classifying persistent PACS patients according to clinical, immunological, and metabolomic profiles.

Our study has several limitations including a small sample size and that the length of time between acute active SARS-CoV-2 infection and the follow-up visit was variable, ranging from 7 to 19 months. This variability in time of sample collection may have influenced some immunological and metabolomic features. Other potential confounders are that we did not have information regarding re-infections between the initial acute infection and the follow-up visit, as well as the vaccination status and the number and type of vaccines received by each subject. Further studies are needed with serial assessment of clinical, immunological, and metabolic features over the time between the acute infection and the development and follow-up of PACS.

## Figures and Tables

**Table 1:** Basal demographic, clinical, and laboratory features in patients with and without persistent PACS.

Variable	Without PACS n = 32	With PACS n = 19	*p*
Demographics			
Age	49.5 years (25–71)	48 years (29–67)	NS
Female gender	12 (37.5%)	7 (36.8%)	NS
Acute infection severity	n (%)	n (%)	
Mild	15 (46.9%)	6 (31.5%)	NS
Moderate/severe	15 (46.9%)	11 (57.8%)	NS
Critical	2 (6.2%)	2 (10.5%)	NS
Clinical/Comorbidities	n (%)	n (%)	
Obesity	6 (18.75%)	9 (47.3%)	NS
Diabetes mellitus	5 (15.6%)	3 (15.78%)	NS
Arterial Hypertension	5 (15.6%)	8 (42.1%)	NS
Cardiopathy	1 (3.12%)	0	NS
Dyslipidemia	0	1 (5.26%)	NS
Chronic renal disease	0	0	NS
Smoking	3 (9.37%)	1 (10.52%)	NS
Laboratory	Median (IQR)	Median (IQR)	
Leucocytes	7150 (5225–8375)	6700 (5200–10,100)	NS
Total T lymphocytes	552 (395–1194)	858 (620–1263)	NS
Ferritin	645 (203–837)	541 (317–1026)	NS
D Dimer	859 (380–1113)	961 (634–1250)	NS

**Table 2:** Basal immunologic and cytokine features in patients with and without persistent PACS.

Cytokines/Chemokines	Without PACS Median (IQR)	With PACS Median (IQR)	*p*
Eotaxin (pg/mL)	104.1 (70.64–111)	80.06 (73.85–102.1)	NS
TGF-β1 (pg/mL)	90,435 (70,347–101,814)	92,251 (79,717–110,959)	NS
TGF-β2 (pg/mL)	2849 (845.3–3435)	1005 (476.1–2775)	NS
TGF-β3 (pg/mL)	6753 (68.24–7444)	716.3 (68.24–5839)	NS
G-CSF (pg/mL)	49.9 (24.93–91.69)	23.39 (9.25–91.69)	NS
IFN-α2 (pg/mL)	27.82 (10.9–44.32)	36.02 (14.81–58.99)	NS
IFN-γ (pg/mL)	11.58 (5.693–20.77)	9.11 (3.05–15)	NS
IL-10 (pg/mL)	15.82 (9.99–23)	17.51 (7.823–24.55)	NS
GM-CSF (pg/mL)	6.79 (4.03–9.19)	7.755 (1.023–10.99)	NS
VEGF (pg/mL)	80.27 (56.33–129.1)	96.3 (58.99–122)	NS
TNF-β (pg/mL)	6.365 (0.7–24.06)	1.945 (0.7–5.04)	NS
TNF-α (pg/mL)	16.88 (12.38–43.71)	33.2 (23.67–134.13)	NS
MIP-1B (pg/mL)	34.45 (22.38–46.78)	43.98 (26.12–66.02)	NS
MIP-1A (pg/mL)	3.88 (0.76–8.513)	2.085 (0.7–11.59)	NS
MCP-1 (pg/mL)	377.9 (222–509.1)	215.8 (94.15–520.8)	NS
IP-10 (pg/mL)	686.7 (421.6–1189)	1344 (498.4–1780)	NS
IL-8 (pg/mL)	17.31 (9.97–31.38)	38.28 (25.84–98.47)	**<0.01**
IL-7 (pg/mL)	14.02 (7.61–34.62)	13.31 (3.49–54.87)	NS
IL-6 (pg/mL)	15.88 (11.96–26.38)	19.43 (6.505–26.97)	NS
IL-5 (pg/mL)	3.47 (1.91–16.91)	6.26 (0.7275–141.3)	NS
IL-4 (pg/mL)	42.26 (11.9–164.9)	11.9 (4.4–34.67)	NS
IL-3 (pg/mL)	0.705 (0.205–0.745)	0.19 (0.145–0.595)	NS
IL-2 (pg/mL)	1.74 (1.09–8.1)	1.89 (0.445–36.29)	NS
IL-1β (pg/mL)	2.93 (1.91–4.05)	4.54 (0.98–6.305)	NS
IL-1A (pg/mL)	11.44 (7.643–36.52)	4.905 (0.98–11.51)	NS
IL-1RA (pg/mL)	36.63 (16.91–53.24)	70.84 (24.18–132.4)	NS
IL-17A (pg/mL)	5.92 (1.833–10.47)	4.62 (1.705–16.89)	NS
IL-15 (pg/mL)	4.78 (3.558–6.805)	3.93 (1.348–6.138)	NS
IL-13 (pg/mL)	5.43 (3.24–9.62)	3.74 (0.56–6.315)	NS
IL-12p70 (pg/mL)	4.63 (1.82–7.333)	4.905 (0.655–14.18)	NS
IL-12p40 (pg/mL)	15.61 (2.95–30.31)	9.095 (2.95–27.9)	NS
IL-18 (pg/mL)	576.2 (333.6–768.8)	693.9 (475.7–1070)	NS
Immune cell subsets	Median (IQR)	Median (IQR)	
B Lymphocytes (cells/μL)	4.24 (2.07–114.8)	54.16 (6.928–107.8)	NS
Memory B cells (cells/μL)	7.965 (0.345–17.99)	7.955 (0.7975–22.25)	NS
Unswitched memory B cells (cells/μL)	0.585 (0.0825–2.98)	1.065 (0.0725–5.583)	NS
Switched memory B cells (cells/μL)	5.975 (0.2625–16)	5.235 (0.395–15.69)	NS
Antibody secreting cells (cells/μL)	0.945 (0.0225–1.453)	0.72 (0.0725–1.518)	NS
CD27- B cells (cells/μL)	43.04 (1.643–73.63)	44.47 (4.36–83.76)	NS
Transitional1 B cells (cells/μL)	0.385 (0.0225–1.978)	0.27 (0.0325–3.348)	NS
Transitional 2 B cells (cells/μL)	0.335 (0.0075–1.723)	1.07 (0.255–4.245)	NS
Mature B cells (cells/μL)	26.52 (1.02–49.47)	29.52 (2.873–61.65)	NS
Double negative B cells (cells/μL)	8.04 (0.27–19.75)	5.275 (0.6875–21.25)	NS
Double negative 1 B cells (cells/μL)	1.24 (0.0375–5.235)	1.87 (0.145–8.72)	NS
Double negative 2 B cells (cells/μL)	0.07 (0–0.605)	0.06 (0–0.81)	NS
Double negative 3 B cells (cells/μL)	4.385 (0.0375–12.5)	2.705 (0.4525–11.25)	NS
Double negative 4 B cells (cells/μL)	0 (0–0.0225)	0 (0–0.0175)	NS
IgD+ B cells (cells/μL)	1.06 (0.1125–3.915)	2.08 (0.14–4.135)	NS
IgD− B cells (cells/μL)	3.74 (0.2025–10.61)	3.065 (0.2975–5.968)	NS
Naïve B cells (cells/μL)	16.89 (0.72–36.38)	20.32 (1.505–41.36)	NS
Resting naïve B cells (cells/μL)	16.6 (0.72–35.93)	19.98 (1.505–41.13)	NS
Activated Naïve B cells (cells/μL)	0.12 (0–0.44)	0.17 (0.005–0.3725)	NS
T1^+^T2 B cells (cells/μL)	1.885 (0.1125–5.72)	1.535 (0.29–8.4)	NS
CD24+CD38^lo-^ (cells/μL)	6.365 (0.33–14.4)	5.195 (0.765–9.09)	NS
CD4+ T cells (cells/μL)	245.5 (139.8–577.2)	274.8 (135.9–312.3)	NS
CD4+ regulatory T cells(cells/μL)	132.7 (50.94–211.3)	99.75 (64.01–201.4)	NS
CD4+ memory T cells(cells/μL)	52.47 (26.9–82.91)	68.11 (38.37–79.54)	NS
CD4+ central memory T cells(cells/μL)	6.995 (1.66–11.68)	4.14 (1.078–7.883)	NS
CD4+ Naïve T cells(cells/μL)	193 (86.22–308.5)	160.6 (73.43–216.4)	NS
CD8+ T cells (cells/μL)	268.4 (93.48–401.2)	175.3 (81.43–375)	NS
MFI of CD57 on CD8+ T cells (cells/μL)	42,308 (11,669–58,972)	55,609 (33,741–85,811)	NS
CD8+ memory T cells (cells/μL)	32.38 (15.43–56.46)	31.25 (19.13–52.1)	NS
CD8+ effector memory T cells (cells/μL)	14.71 (8.048–33.88)	18.98 (9.748–33.37)	NS
CD8+ central memory T cells (cells/μL)	1.51 (0.55–5.755)	2.115 (0.515–2.408)	NS
Naïve CD8+ T cells (cells/μL)	196.4 (73.71–233.6)	115.2 (61.23–267.3)	NS
Th1 cells (cells/μL)	65.2 (23.7–165.9)	109.1 (63.39–180.7)	NS
Th2 cells (cells/μL)	10.26 (4.555–25.44)	10.72 (3.608–21)	NS
Th17 cells (cells/μL)	1.66 (0.3625–6.973)	2.395 (0.8125–4.913)	NS
Tc1 cells (cells/μL)	78.01 (25.47–266.8)	143.8 (53.98–251.6)	NS
Tc2 cells (cells/μL)	3.66 (2.265–9.193)	3.76 (1.713–8.678)	NS
Tc17 cells (cells/μL)	3.66 (2.265–9.193)	3.76 (1.713–8.678)	NS
Classical monocytes (cells/μL)	306.1 (251.4–400.5)	338.2 (189.9–526.6)	NS
Intermediate monocytes (cells/μL)	53.04 (19.75–127.6)	60.13 (29.06–125.7)	NS
Non-classical monocytes (cells/μL)	21.73 (12.77–46.74)	37.9 (20.8–99.57)	NS
Immature low density granulocytes (%)	0.09 (0.035–0.3025)	0.12 (0.01–0.965)	NS
Immature low density granulocytes (cells/μL)	0.055 (0.01–0.5775)	0.26 (0–1.11)	NS
Mature low density granulocytes (%)	0.55 (0.32–1.133)	1.555 (0.5725–4.268)	**<0.02**
Mature low density granulocytes (cells/μL)	0.225 (0.0675–1.403)	1.85 (0.17–13.43)	NS
NK cells (cells/μL)	19.65 (10.35–29.88)	20.35 (7.195–31.73)	NS
CD56^high^ cells (cells/μL)	7.725 (4.653–12.63)	5 (2.25–8.573)	NS
CD56^lo^ cells (cells/μL)	92 (87–95.3)	95 (91.4–97.75)	NS

TGF = transforming growth factor; G-CSF = granulocyte colony-stimulating factor; IFN = interferon; IL = interleukin; GM-CSF = granulocyte–macrophage colony-stimulating factor; VEGF = vascular endothelial growth factor; TNF = tumor necrosis factor; MIP = macrophage inflammatory protein; MCP = monocyte chemoattractant protein; IP = IFNγ-induced protein; NK = natural killer; MFI = mean fluorescence intensity; IQR = interquartilar range. Values in bold reflect statistical significance.

**Table 3:** Basal metabolomic features in patients with and without persistent PACS.

Metabolites	Without PACS Median (IQR)	With PACS Median (IQR)	*p*
Pyruvate	1.844 (0.6563–2.701)	3.114 (2.193–5.362)	**<0.02**
Glycolic acid	0.4015 (0.368–0.5598)	0.3815 (0.3462–0.5491)	NS
2-keto-3-methylvaleric acid	3.652 (3.191–4.3)	3.528 (2.981–4.513)	NS
Alpha-hydroybutyric acid	28.14 (18.52–48.82)	42.28 (24.31–51.76)	NS
3-hydroxybutyric acid	8.195 (4.863–18.34)	6.419 (4.195–10.72)	NS
Alpha-hydroxyisovaleric acid	4.568 (3.899–8.757)	6.99 (5.424–21.35)	NS
Beta-alanine	1.724 (1.403–2.418)	1.482 (1.272–2.127)	NS
3-hydroxyisovaleric acid	1.741 (1.413–2.201)	1.967 (1.453–3.456)	NS
Valine 2TMS	33.76 (30.54–37.76)	34.87 (28.52–37.73)	NS
Leucine 2TMS	18.87 (15.09–20.17)	19.54 (15.33–20.74)	NS
Glycerol	19.02 (12.98–29.34)	15.59 (12.62–26.14)	NS
Isoleucine 2TMS	58.57 (48.28–69.39)	60.33 (36.31–80.24)	NS
Proline 2TMS	24.04 (16.87–29.08)	20.31 (15.51–25.4)	NS
Pipecolinic acid	2.066 (1.595–2.792)	1.152 (0.9851–2.096)	NS
Glyceric acid	0.1139 (0.0891–0.1349)	0.1008 (0.07092–0.1594)	NS
2,3-dihydroxybutanoic acid	1.194 (0.8146–2.5)	0.8958 (0.6935–1.022)	NS
Serine 3TMS	70.22 (64.25–79.05)	71.28 (61.78–81.82)	NS
Threonine 3TMS	54.97 (48.77–73.67)	50.47 (43.86–75.71)	NS
3,4-dihydroxybutanoic acid	2.01 (1.692–2.523)	2.921 (1.839–3.793)	NS
Malic acid	2.98 (2.399–4.069)	4.309 (2.325–5.804)	NS
Methionine 2TMS	2.321 (2.097–2.515)	2.557 (1.805–2.652)	NS
5-oxoproline	62.35 (54.03–65.8)	51.65 (51–76.96)	NS
Cysteine 3TMS	6.729 (5.233–8.265)	8.384 (6.411–12.62)	NS
Threonic acid	6.675 (3.999–8.895)	8.187 (4.092–12.45)	NS
Alpha-ketoglutarate	4.363 (3.34–5.842)	5.434 (3.878–7.548)	NS
Ornithine	3.672 (2.549–4.483)	3.941 (3.299–6.685)	NS
Glutamic acid 3TMS	43.78 (30.03–60.35)	56.74 (40.73–71.63)	NS
Phenylalanine 2TMS	40.87 (32.91–48.38)	40.6 (37.75–45.18)	NS
Lysine 3TMS	26.42 (17.57–36.95)	32.54 (21.06–44.83)	NS
Glutamine 3TMS	74.46 (66.08–85.97)	69.38 (38.27–83.38)	NS
Azelaic acid	8.829 (5.808–12.17)	8.512 (3.258–11.68)	NS
hypoxanthine	4.466 (3.74–9.376)	4.081 (3.211–4.76)	NS
Ornithine	15.91 (11.71–25.67)	21.73 (13.56–23.67)	NS
Citric acid	7.113 (4.643–9.788)	4.943 (4.249–6.16)	NS
Myristic acid	3.323 (2.426–4.9)	2.853 (2.376–4.483)	NS
1,5-anhydro-D-sorbitol	62.49 (52.08–82.1)	56.85 (29.94–93.65)	NS
Tyrosine 3TMS	59.48 (51.92–76.6)	60.75 (49.37–71.57)	NS
Palmitic acid	68.83 (56.72–85.56)	71.35 (62.74–90.55)	NS
Myo-inositol	12.78 (10.59–17.97)	14.37 (11.34–16.28)	NS
Heptadecanoic acid	1.147 (0.9246–1.288)	1.018 (0.6353–1.086)	NS
Oleic acid	3.75 (2.69–6.138)	4.872 (3.724–9.967)	NS
Stearic acid	51.3 (46.13–56.93)	51.46 (41.07–54.04)	NS
Cystine	9.655 (3.803–14.16)	19.13 (17.18–21.08)	**<0.01**
Pseudouridine	1.575 (1.44–1.929)	1.729 (1.441–2.419)	**<0.04**
Alpha-tocopherol	4.663 (3.536–7.073)	3.499 (2.674–3.674)	NS
Cholesterol	25 (22.4–27.19)	23.31 (18.9–26.24)	NS

Values in bold reflect statistical significance.

**Table 4:** Baseline variables associated with persistent PACS in univariate analysis.

Variable	OR	95% CI	*p* Value
Body mass index	1.15	1.00–1.35	0.04
ALT	0.99	0.98–0.99	0.01
Pyruvate	1.83	1.09–3.64	0.01
Cystine	1.15	1.02–1.36	0.01
Alpha tocopherol	0.59	0.30–0.98	0.04
Albumine	0.42	0.12–1.13	0.09
MIP 1b	1.02	0.99–1.06	0.09
IL-8	1.00	0.99–1.01	0.09
Pipecolinic acid	0.30	0.65–1.04	0.058
2,3 dyhidroxybutanoic acid	0.43	0.11–1.05	0.06
Malic acid	1.71	0.97–3.80	0.06
Cysteine 3 TMS	1.36	0.98–2.05	0.059
Heptadecanoic acid	0.05	0.001–1.40	0.08
Absolute B lymphocytes TR2+	1.30	0.96–2.16	0.09
NK lymphocytes CD56^low^	1.15	0.97–1.41	0.09

## Data Availability

Additional data are available upon request from the corresponding author.

## Supplementary Materials

The following supporting information can be downloaded at: https://www.mdpi.com/article/10.3390/ijms25179661/s1.
