# Supplementary material for: Novel Clinical, Immunological, and Metabolic Features Associated with Persistent Post-Acute COVID-19 Syndrome
Source: Int J Mol Sci. 2024 Sep 6;25(17):9661. doi: 10.3390/ijms25179661 (PMC11395921; doi:10.3390/ijms25179661)

Supplements:

Supplementary Table S1. Most frequent PACS symptoms.

Symptom	Without PACS n=32	With PACS n=19
Confusion	NA	4 (21%)
Cough	NA	7 (36.8%)
Dysnea	NA	7 (36.8%)
Headache	NA	11(57.8%)
Arthralgias	NA	9 (47.3%)
Myalgias	NA	4 (21%)
Cutaneous rash	NA	3 (15.7%)

NA: Not applicable, PACS = persistent acute COVID syndrome

Supplementary Figure S1.

A) Gating strategy for the immunotipification of T cell subsets.

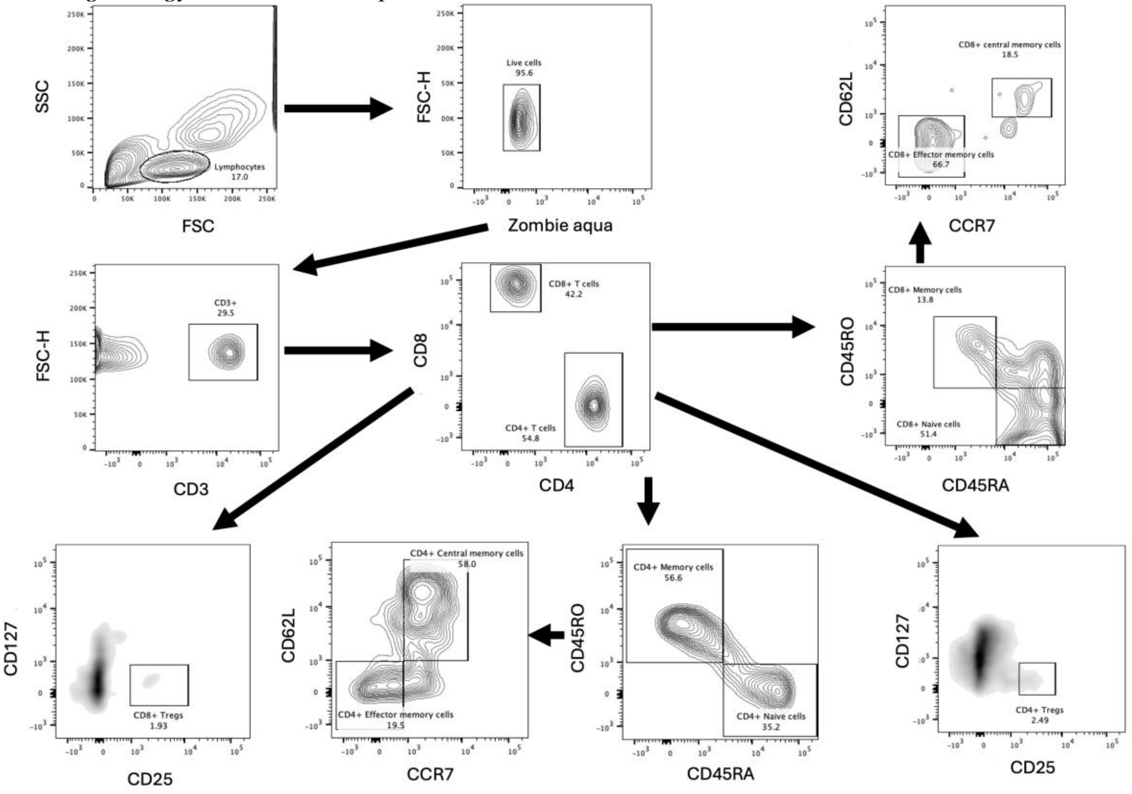

B) Gating strategy for the immunotipification of B cell subsets

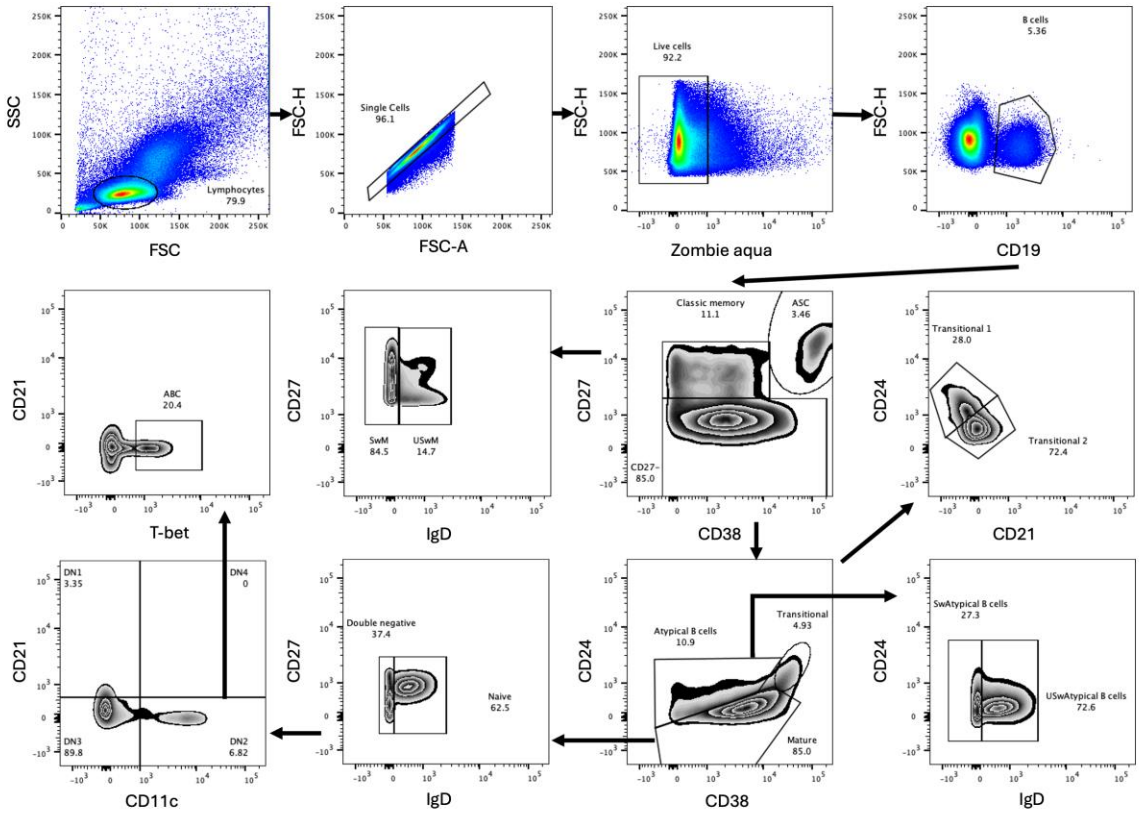

C) Gating strategy for the identification of Th and Tc subsets

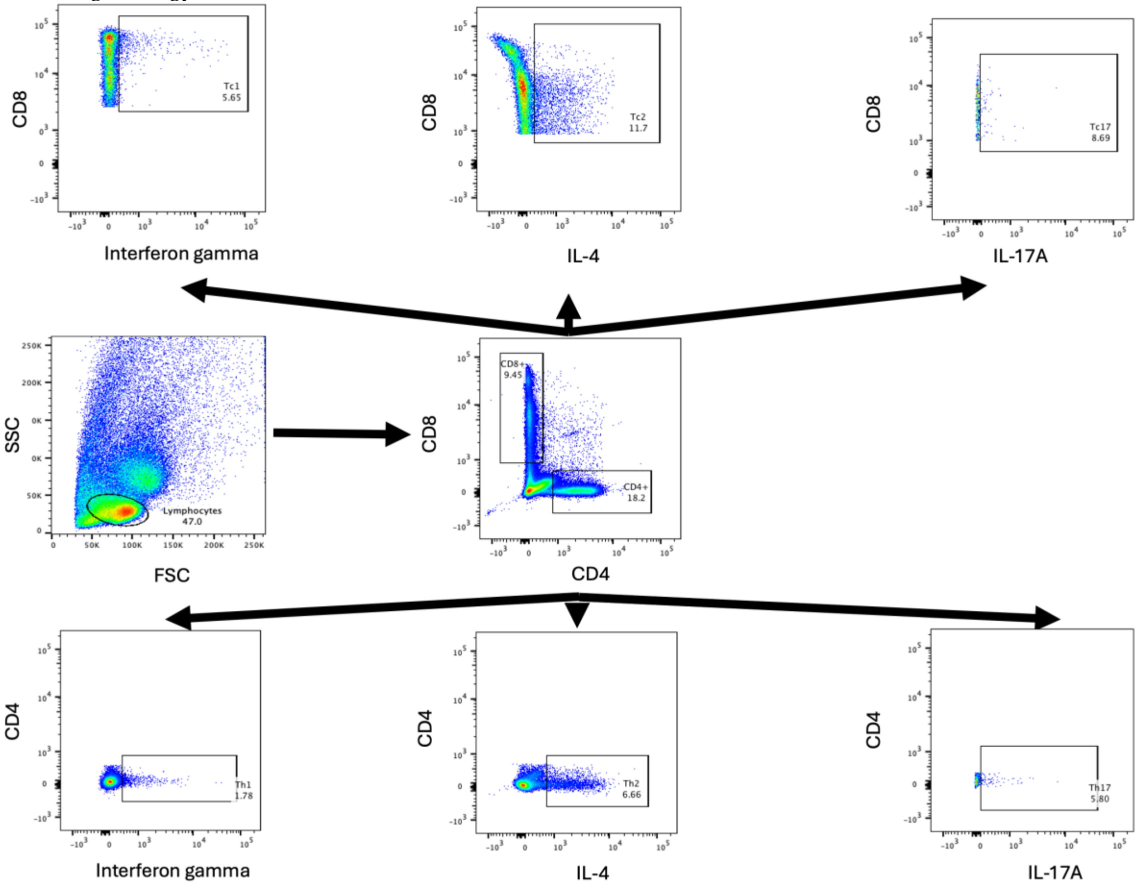

D) Gating strategy for the quantification of the proportion of exhausted, anergic and senescent T cells

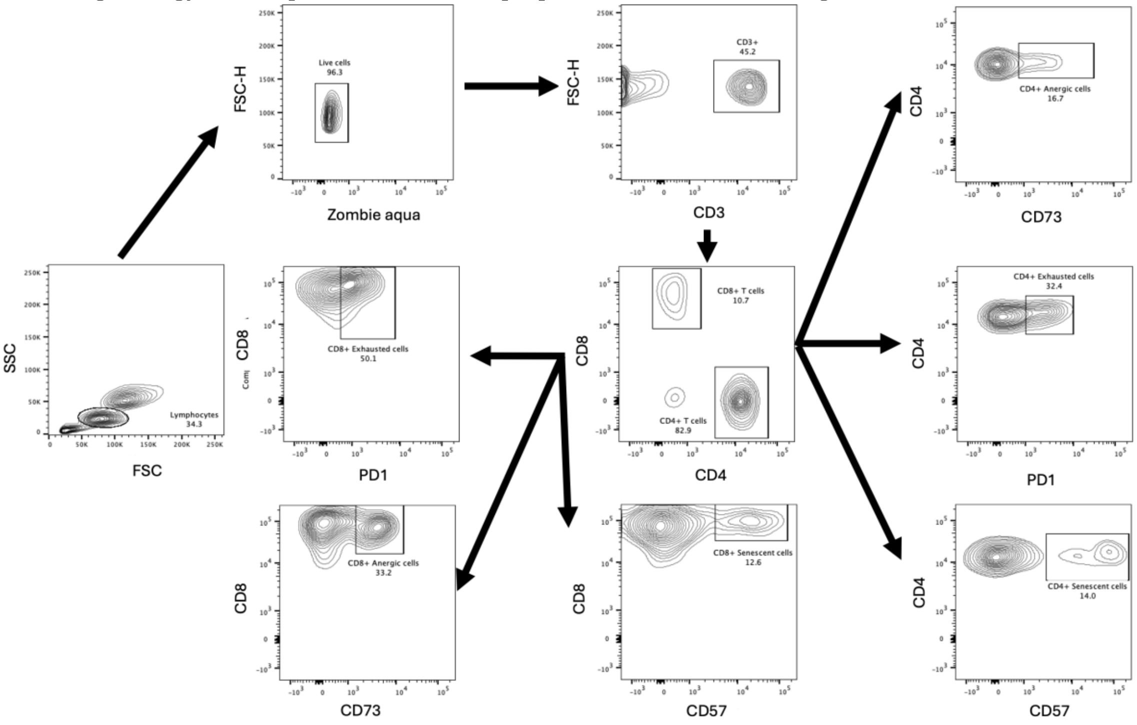

E) Gating strategy for the detection of low-density granulocytes and monocyte subsets

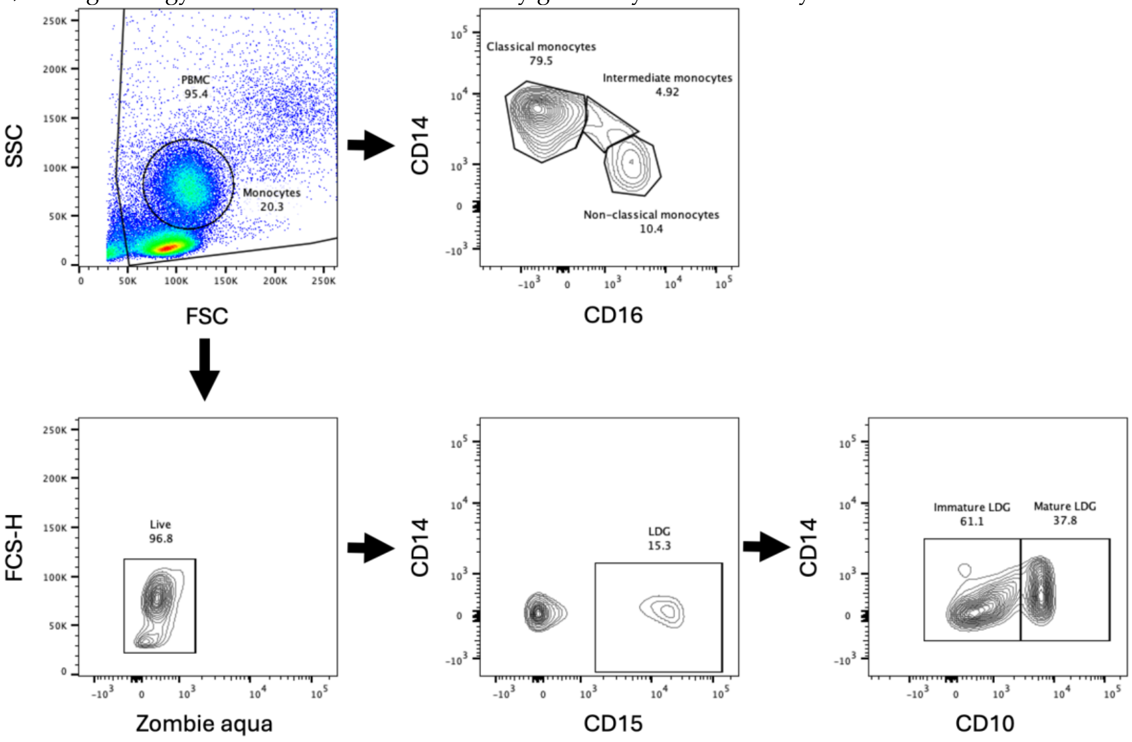

Supplement: Supplementary file 1 [file ijms-25-09661-s001.zip › ijms-3180158-supplementary.pdf]
